# Real‐world data using mHealth apps in rhinitis, rhinosinusitis and their multimorbidities

**DOI:** 10.1002/clt2.12208

**Published:** 2022-11-18

**Authors:** Bernardo Sousa‐Pinto, Aram Anto, Markus Berger, Stephanie Dramburg, Oliver Pfaar, Ludger Klimek, Marek Jutel, Wienczyslawa Czarlewski, Anna Bedbrook, Arunas Valiulis, Ioana Agache, Rita Amaral, Ignacio J. Ansotegui, Katharina Bastl, Uwe Berger, Karl C. Bergmann, Sinthia Bosnic‐Anticevich, Fulvio Braido, Luisa Brussino, Victoria Cardona, Thomas Casale, G. Walter Canonica, Lorenzo Cecchi, Denis Charpin, Tomás Chivato, Derek K. Chu, Cemal Cingi, Elisio M. Costa, Alvaro A. Cruz, Philippe Devillier, Stephen R. Durham, Motohiro Ebisawa, Alessandro Fiocchi, Wytske J. Fokkens, Bilun Gemicioğlu, Maia Gotua, Maria‐Antonieta Guzmán, Tari Haahtela, Juan Carlos Ivancevich, Piotr Kuna, Igor Kaidashev, Musa Khaitov, Violeta Kvedariene, Désirée E. Larenas‐Linnemann, Brian Lipworth, Daniel Laune, Paolo M. Matricardi, Mario Morais‐Almeida, Joaquim Mullol, Robert Naclerio, Hugo Neffen, Kristoff Nekam, Marek Niedoszytko, Yoshitaka Okamoto, Nikolaos G. Papadopoulos, Hae‐Sim Park, Giovanni Passalacqua, Vincenzo Patella, Simone Pelosi, Nhân Pham‐Thi, Ted A. Popov, Frederico S. Regateiro, Sietze Reitsma, Monica Rodriguez‐Gonzales, Nelson Rosario, Philip W. Rouadi, Boleslaw Samolinski, Ana Sá‐Sousa, Joaquin Sastre, Aziz Sheikh, Charlotte Suppli Ulrik, Luis Taborda‐Barata, Ana Todo‐Bom, Peter Valentin Tomazic, Sanna Toppila‐Salmi, Salvatore Tripodi, Ioanna Tsiligianni, Erkka Valovirta, Maria Teresa Ventura, Antonio A. Valero, Rafael José Vieira, Dana Wallace, Susan Waserman, Sian Williams, Arzu Yorgancioglu, Luo Zhang, Mihaela Zidarn, Jaron Zuberbier, Heidi Olze, Josep M. Antó, Torsten Zuberbier, João A. Fonseca, Jean Bousquet

**Affiliations:** ^1^ MEDCIDS ‐ Department of Community Medicine, Information and Health Decision Sciences Faculty of Medicine University of Porto Porto Portugal; ^2^ CINTESIS – Center for Health Technology and Services Research, University of Porto Porto Portugal; ^3^ RISE – Health Research Network University of Porto Porto Portugal; ^4^ MASK‐air Montpellier France; ^5^ Department of Pathophysiology and Allergy Research Center for Pathophysiology, Infectiology and Immunology Medical University of Vienna Vienna Austria; ^6^ Department for Oto‐Rhino‐Laryngology, Head and Neck Surgery Medical University of Vienna Vienna Austria; ^7^ Pediatric Pulmonology, Immunology and Intensive Care Medicine Charité Universitätsmedizin Berlin Berlin Germany; ^8^ Department of Otorhinolaryngology, Head and Neck Surgery Section of Rhinology and Allergy University Hospital Marburg Philipps‐Universität Marburg Marburg Germany; ^9^ Department of Otolaryngology, Head and Neck Surgery Universitätsmedizin Mainz Mainz Germany; ^10^ Center for Rhinology and Allergology Wiesbaden Germany; ^11^ Department of Clinical Immunology Wrocław Medical University ALL‐MED Medical Research Institute Wroclaw Poland; ^12^ Medical Consulting Czarlewski Levallois France; ^13^ Fraunhofer Institute for Translational Medicine and Pharmacology ITMP, Allergology and Immunology Berlin Germany; ^14^ Institute of Clinical Medicine and Institute of Health Sciences Medical Faculty of Vilnius University Vilnius Lithuania; ^15^ Transylvania University Brasov Brasov Romania; ^16^ Department of Allergy and Immunology Hospital Quirónsalud Bizkaia Bilbao Spain; ^17^ Institute of Allergology Charité – Universitätsmedizin Berlin Corporate Member of Freie Universität Berlin and Humboldt‐Universität zu Berlin Berlin Germany; ^18^ Quality Use of Respiratory Medicine Group Woolcock Institute of Medical Research The University of Sydney Sydney New South Wales Australia; ^19^ Department of Internal Medicine (DiMI), University of Genoa IRCCS Ospedale Policlinico San Martino Genova Italy; ^20^ Department of Medical Sciences Allergy and Clinical Immunology Unit University of Torino & Mauriziano Hospital Torino Italy; ^21^ Allergy Section Department of Internal Medicine Hospital Vall d'Hebron & ARADyAL Research Network Barcelona Spain; ^22^ Division of Allergy/immunology University of South Florida Tampa Florida USA; ^23^ Department of Biomedical Sciences Humanitas University Pieve Emanuele, Milan & Personalized Medicine, Asthma and Allergy, Humanitas Clinical and Research Center IRCCS Rozzano Italy; ^24^ SOS Allergology and Clinical Immunology USL Toscana Centro Prato Italy; ^25^ Clinique des bronches, allergie et sommeil Hôpital Nord Marseille France; ^26^ School of Medicine University CEU San Pablo Madrid Spain; ^27^ Department of Health Research Methods, Evidence and Impact & Department of Medicine McMaster University Hamilton Ontario Canada; ^28^ Eskisehir Osmangazi University Medical Faculty ENT Department Eskisehir Turkey; ^29^ UCIBIO REQUINTE Faculty of Pharmacy and Competence Center on Active and Healthy Ageing of University of Porto (Porto4Ageing) Porto Portugal; ^30^ Fundaçao ProAR Federal University of Bahia and GARD/WHO Planning Group Salvador Bahia Brazil; ^31^ VIM Suresnes, UMR 0892, Pôle des Maladies des Voies Respiratoires, Hôpital Foch Université Paris‐Saclay Suresnes France; ^32^ Allergy and Clinical Immunology National Heart and Lung Institute Imperial College London London UK; ^33^ Clinical Research Center for Allergy and Rheumatology NHO Sagamihara National Hospital Sagamihara Japan; ^34^ Division of Allergy Department of Pediatric Medicine ‐ The Bambino Gesù Children's Research Hospital IRCCS Rome Italy; ^35^ Department of Otorhinolaryngology Amsterdam University Medical Centres Amsterdam The Netherlands; ^36^ Department of Pulmonary Diseases Istanbul University‐Cerrahpasa Cerrahpasa Faculty of Medicine Istanbul Turkey; ^37^ Center of Allergy and Immunology Georgian Association of Allergology and Clinical Immunology Tbilisi Georgia; ^38^ Immunology and Allergy Division Clinical Hospital University of Chile Santiago Chile; ^39^ Skin and Allergy Hospital Helsinki University Hospital University of Helsinki Helsinki Finland; ^40^ Servicio de Alergia e Immunologia Clinica Santa Isabel Buenos Aires Argentina; ^41^ Division of Internal Medicine, Asthma and Allergy Barlicki University Hospital Medical University of Lodz Lodz Poland; ^42^ Poltava State Medical University Poltava Ukraine; ^43^ National Research Center Institute of Immunology Federal Medicobiological Agency Laboratory of Molecular Immunology Moscow Russia; ^44^ Pirogov Russian National Research Medical University Moscow Russia; ^45^ Institute of Biomedical Sciences Department of Pathology Faculty of Medicine Vilnius University and Institute of Clinical Medicine, Clinic of Chest Diseases and Allergology, Faculty of Medicine, Vilnius University Vilnius Lithuania; ^46^ Center of Excellence in Asthma and Allergy Médica Sur Clinical Foundation and Hospital México City Mexico; ^47^ Scottish Centre for Respiratory Research Cardiovascular & Diabetes Medicine Medical Research Institute Ninewells Hospital University of Dundee Dundee UK; ^48^ KYomed INNOV Montpellier France; ^49^ Allergy Center CUF Descobertas Hospital Lisbon Portugal; ^50^ Rhinology Unit & Smell Clinic ENT Department Hospital Clínic and Clinical & Experimental Respiratory Immunoallergy, IDIBAPS, CIBERES, University of Barcelona Barcelona Spain; ^51^ Johns Hopkins School of Medicine Baltimore Maryland USA; ^52^ Director of Center of Allergy, Immunology and Respiratory Diseases Santa Fe Argentina; ^53^ Hospital of the Hospitaller Brothers in Buda Budapest Hungary; ^54^ Department of Allergology Medical University of Gdańsk Gdańsk Poland; ^55^ Chiba University Hospital and Chiba Rosai Hospital Chiba Japan; ^56^ Allergy Department 2nd Pediatric Clinic University of Athens Athens Greece; ^57^ Department of Allergy and Clinical Immunology Ajou University School of Medicine Suwon South Korea; ^58^ Allergy and Respiratory Diseases IRCCS Polyclinic Hospital San Martino University of Genoa Genova Italy; ^59^ Division of Allergy and Clinical Immunology, Department of Medicine "Santa Maria della Speranza" Hospital, Battipaglia and Agency of Health ASL Salerno Italy; ^60^ TPS Production Rome Italy; ^61^ Ecole Polytechnique Palaiseau IRBA (Institut de Recherche bio‐Médicale des Armées) Bretigny France; ^62^ University Hospital 'Sv Ivan Rilski' Sofia Bulgaria; ^63^ Allergy and Clinical Immunology Unit Centro Hospitalar e Universitário de Coimbra Coimbra and Institute of Immunology Faculty of Medicine University of Coimbra Coimbra Portugal; ^64^ Coimbra Institute for Clinical and Biomedical Research (iCBR) Faculty of Medicine University of Coimbra Coimbra Portugal; ^65^ Pediatric Allergy and Clinical Immunology Hospital Español de Mexico Mexico City Mexico; ^66^ Hospital de Clinicas University of Parana Umuarama Brazil; ^67^ Department of Otolaryngology‐Head and Neck Surgery Eye and Ear University Hospital Beirut Lebanon; ^68^ Department of Otorhinolaryngology‐Head and Neck Surgery Dar Al Shifa Hospital Salmiya Kuwait; ^69^ Department of Prevention of Environmental Hazards, Allergology and Immunology Medical University of Warsaw Warsaw Poland; ^70^ Fundacion Jimenez Diaz, CIBERES Faculty of Medicine Autonoma University of Madrid Madrid Spain; ^71^ Usher Institute The University of Edinburgh Edinburgh UK; ^72^ Department of Respiratory Medicine Copenhagen University Hospital‐Hvidovre Copenhagen Denmark; ^73^ Institute of Cinical Medicine University of Copenhagen Copenhagen Denmark; ^74^ Department of Immunoallergology, Cova da Beira University Hospital Centre and UBIAir ‐ Clinical & Experimental Lung Centre and CICS‐UBI Health Sciences Research Centre University of Beira Interior Covilhã Portugal; ^75^ Department of General ORL, H&NS Medical University of Graz ENT‐University Hospital Graz Graz Austria; ^76^ Allergy Unit Policlinico Casilino Rome Italy; ^77^ Health Planning Unit Department of Social Medicine Faculty of Medicine University of Crete Greece and International Primary Care Respiratory Group IPCRG Aberdeen Scotland; ^78^ Department of Lung Diseases and Clinical Immunology University of Turku and Terveystalo Allergy Clinic Turku Finland; ^79^ Unit of Geriatric Immunoallergology University of Bari Medical School Bari Italy; ^80^ Pneumology and Allergy Department CIBERES and Clinical & Experimental Respiratory Immunoallergy IDIBAPS University of Barcelona Barcelona Spain; ^81^ Nova Southeastern University Fort Lauderdale Florida USA; ^82^ Department of Medicine, Clinical Immunology and Allergy McMaster University Hamilton Ontario Canada; ^83^ International Primary Care Respiratory Group IPCRG Larbert Scotland; ^84^ Department of Pulmonary Diseases Celal Bayar University, Faculty of Medicine Manisa Turkey; ^85^ Department of Otolaryngology Head and Neck Surgery Beijing TongRen Hospital and Beijing Institute of Otolaryngology Beijing China; ^86^ University Clinic of Respiratory and Allergic Diseases Golnik Slovenia; ^87^ University of Ljubljana Faculty of Medicine Ljubljana Slovenia; ^88^ Department of Otorhinolaryngology Charité‐Universitätsmedizin Berlin Berlin Germany; ^89^ ISGlobal, Barcelona Institute for Global Health Barcelona Spain; ^90^ IMIM (Hospital del Mar Medical Research Institute) Barcelona Spain; ^91^ Universitat Pompeu Fabra (UPF) Barcelona Spain; ^92^ CIBER Epidemiología y Salud Pública (CIBERESP) Barcelona Spain; ^93^ University Hospital Montpellier Montpellier France

**Keywords:** allergic rhinitis, app, chronic rhinosinusitis, mHealth

## Abstract

Digital health is an umbrella term which encompasses eHealth and benefits from areas such as advanced computer sciences. eHealth includes mHealth apps, which offer the potential to redesign aspects of healthcare delivery. The capacity of apps to collect large amounts of longitudinal, real‐time, real‐world data enables the progression of biomedical knowledge. Apps for rhinitis and rhinosinusitis were searched for in the Google Play and Apple App stores, via an automatic market research tool recently developed using JavaScript. Over 1500 apps for allergic rhinitis and rhinosinusitis were identified, some dealing with multimorbidity. However, only six apps for rhinitis (AirRater, AllergyMonitor, AllerSearch, Husteblume, MASK‐air and Pollen App) and one for rhinosinusitis (Galenus Health) have so far published results in the scientific literature. These apps were reviewed for their validation, discovery of novel allergy phenotypes, optimisation of identifying the pollen season, novel approaches in diagnosis and management (pharmacotherapy and allergen immunotherapy) as well as adherence to treatment. Published evidence demonstrates the potential of mobile health apps to advance in the characterisation, diagnosis and management of rhinitis and rhinosinusitis patients.

## 1 INTRODUCTION

The burden and cost of allergic and chronic respiratory diseases are increasing worldwide, with most economies struggling to effectively respond.^1^, ^2^, ^3^, ^4^ Transforming healthcare systems requires strengthened integrated care using organisational health literacy. For this, digital health may be particularly useful, as it may put the patient at the centre of his/her disease management, promote better monitoring and improve patient education. This is particularly true for non‐communicable diseases, whose burden is expected to increase in the near future. It is therefore essential to know of the available digital health tools for each disease and how can they be further explored to improve their management.

Digital health is an umbrella term which encompasses eHealth and benefits from areas such as advanced computer sciences (e.g., ‘big data’ and artificial intelligence). eHealth, as defined by the World Health Organization (WHO),^5^ comprises several components including electronic health records, telehealth and mobile health (mHealth). The latter has been defined as a ‘medical and public health practice supported by mobile devices, such as mobile phones’.^6^ It includes: (i) equipment/connected medical devices, (ii) mHealth services and (iii) mHealth apps.^7^, ^8^

Apps designed for and used in allergic rhinitis (AR) and chronic rhinosinusitis (CRS) may help to better understand these diseases and their management as well as to identify and address some unmet needs. This is particularly important in these chronic diseases which are often trivialised^9^ and undertreated,^10^, ^11^ both by patients and healthcare providers. However, these new tools first need to be tested for privacy rules, acceptability, usability and cost‐effectiveness. In addition, they should be evaluated for their impact on (i) the digital transformation of health, (ii) healthcare delivery and (iii) health outcomes. Given the potential of mHealth tools to enable the digital transformation of health and care, empowering citizens and building a healthier society,^12^ it is of great importance to review apps whose data collection tools (e.g., questionnaires) have been validated for the case study chronic conditions of allergic rhinitis (AR) and CRS.

In the present paper, all apps relevant to AR and CRS management retrieved using a market research tool based on an automatic search process will be presented. However, only apps with peer‐reviewed published data for a given disease will be reviewed. The application of these tools/apps will be discussed regarding their potential for identifying disease phenotypes based on real‐life direct patient‐centred data, diagnosis, management and adherence to treatment, as well as for promoting the digital transformation of health and care.

## 2 MARKET RESEARCH FOR MHEALTH APPS IN RHINOLOGY

### 2.1 Identification of mHealth apps

An important challenge for app review studies concerns the lack of automatic standardised search strategies, rendering the identification of potentially relevant apps a time‐consuming manual task.^13^ Such limitations could be overcome by the development of automatic methods for app screening. Recently, such methods have been described for breast cancer,^14^ AR,^15^ urticaria^16^ and anaphylaxis^8^ apps. Automatic methods for app screening also have the advantage of running screening processes more frequently than manual approaches and at an increased speed, and of potentially identifying relevant apps whose name and icon are not obvious.

The method used for the identification of relevant mHealth apps in rhinitis has been described elsewhere. In this review, we will focus on (i) the four apps identified by that study as having associated scientific publications for AR^15^ as (ii) two additional apps for which scientific publications were subsequently identified. In brief, an app screening programme capable of performing searches in app stores without any human intervention has been developed for searching for AR apps using JavaScript,^15^, ^17^ a commonly used programming language that allows searches of dynamic content on web pages.^18^ The screening programme builds upon two open‐source packages.^19^

On the other hand, relevant apps in CRS had not been previously identified. In this study, we used the aforementioned app screening programme to scrape Apple App and Google Play stores^20^ for searching for CRS apps, according to the following criteria: (i) search terms: rhinitis, hay fever, rhinosinusitis; sinusitis; (ii) countries: United Kingdom, United States, Germany (since we wanted to have two English‐speaking countries plus another one with a different language); and (iii) languages: English, German. The number of results retrieved at each iteration was limited to a maximum of 200 apps for Apple/iOS or Android. After retrieving the search results from all iterations, the programme compared the names of all of the retrieved apps and discarded duplicates. A PubMed search up to November 2021 on the names of the retrieved relevant apps was then carried out to identify published peer‐reviewed papers on all such apps.

### 2.2 Allergic rhinitis and CRS

Using automatic and manual search methods, we identified six relevant apps for AR for which data have been published in the literature: AllergyMonitor^®^,^21^, ^22^, ^23^, ^24^, ^25^ AirRater^®^,^26^ MASK‐air^®^,^12^, ^27^, ^28^, ^29^, ^30^, ^31^, ^32^, ^33^, ^34^, ^35^, ^36^, ^37^, ^38^, ^39^, ^40^, ^41^, ^42^, ^43^, ^44^, ^45^ Pollen App (patient's hay fever diary, developed in Austria),^46^, ^47^, ^48^ Husteblume (a mobile phone health app developed in Germany as a spin‐off of Pollen App including the patient hay fever diary) and AllerSearch (Table 1).^55^ Galenus Health did not have any published data on AR and could therefore not be considered in this section for AR, but will be discussed in the subsection of CRS apps.

**TABLE 1:** Apps relevant for allergic rhinitis and rhinosinusitis management and with published data on rhinitis

	Allergy monitor	AirRater	MASK‐air	Pollen	Husteblume	AllerSearch	Galenus health
Countries	14	1	28	7	1	1	?, the current version is not yet available in all countries
Last update	May 2021	Jun 2021	Mar 2021	Nov 2020	Apr 2021	Jan 2022	Jan 2021
Published methodological or clinical validation	^21^, ^22^, ^24^, ^25^, ^49^, ^50^, ^51^	^26^, ^52^, ^53^	^12^, ^27^, ^28^, ^29^, ^30^, ^31^, ^32^, ^33^, ^34^, ^35^, ^36^, ^37^, ^38^, ^39^, ^40^, ^41^	^48^, ^54^	^55^	^56^, ^57^	For rhinosinusitis only, not for rhinitis^58^, ^59^
List of all medications	YES, by medication and dosage, customised by country	YES	YES, by medication, customised by country	YES, by drug class	YES	NOT in daily questionnaires	YES
Published general data protection regulation (GDPR)	YES	NO	YES^60^, ^61^	NO	NO	NO	NO

Among the 17 apps retrieved for CRS, only one had published data for this disease, namely Galenus Health (with a single identified paper) (Table 2). However, in this paper, the app is labelled as MySinusitisCoach, developed using the MASK‐air^®^ structure.^58^ A new app designed by members of the Mayo Clinic has been developed, but solely tested in a pilot study assessing 10 participants.^62^ Kagen Air was only presented in a review paper and was therefore excluded.^63^

**TABLE 2:** Apps retrieved by an automatic search for chronic rhinosinusitis

App name	Availability
1. Sinus Infection Symptoms	Google
2. Headache Tracker ‐ Migraine & Headache Log	Google
3. Ada – check your health	Google
4. Symptomate – Symptom checker	Google
5. Migraine and headache diary	Google
6. Feeble: symptom and illness tracker	Google
7. Headache Log	Google
8. MeMD – Doctor’s Visits Online!	Google
9. Allergy, Asthma & Sinus Center	Apple
10. SinusMonitor	Apple
11. KagenAir	Apple
12. Correlate: Health Journal	Apple
13. MigraineMind Migraine Diary	Apple
14. iGeoPolen Portugal	Apple
15. PROMinENT ‐ Reporting System	Apple
16. Galenus Health	Google, Apple
17. Docquity‐ The Doctors' Network	Google

### 2.3 Limitations of the followed approaches

We used a small set of search terms, which may correspond to a restrictive approach. Nevertheless, these terms were chosen by the Allergic Rhinitis and its Impact on Asthma (ARIA) expert group. Moreover, in performing a PubMed search for ‘apps’, ‘mHealth’, ‘eHealth’ AND ‘rhinitis’ or ‘rhinosinusitis’, we did not find any other apps other than the ones included in this review. Only two languages were searched and some apps may exist in other countries (e.g., in Polish, the *Apsik* and *Dzienniki Alergika* apps are available). However, only one app has been described in articles available in PubMed.^55^ An additional limitation is that some apps may not have used the name they are currently using. This is, for example, the case for Galenus Health which was labelled MySinusitisCoach.^58^ Finally, we did not search any other medical literature databases and may have missed some apps.

## 3 VALIDATION AND MAIN CHARACTERISTICS OF mHEALTH APPS IN RHINOLOGY

### 3.1 Allergic rhinitis

#### 3.1.1 AllergyMonitor^®^

AllergyMonitor^®^ (TPS software production, Rome, Italy) is an online service that was developed in 2009 with the aim of (i) enabling the recording of clinical symptoms, drug use and adherence to allergen immunotherapy (AIT) and (ii) monitoring the efficacy of sublingual or subcutaneous AIT by patients with allergic rhino‐conjunctivitis and/or asthma. The system, available to everyone and simple to use, consists of two parts: a patient app (front end) and a website for the attending doctor (back‐office).^21^ The results can be accessed by the patient and attending physician—as concise reports via a smartphone or computer—in a collaborative setting of blended care. Geolocation is optional.

The download and usage of this app are free of charge and there are no advertisements. It falls under Italian jurisdiction, is CE1 registered and follows the General Data Protection Regulation (GDPR). The Technology Readiness Level (TRL) has been assessed for this app.^64^ It is available in 14 countries (TRL9) and in several languages. It contains the Control of Allergic Rhinitis and Asthma Test (CARAT) as well as pollen counts (TRL9).

The quality of the AllergyMonitor^®^ data was checked by estimating the percentage of changes in trends of the trajectories produced by the patients' data.^49^

##### Methods for statistical analysis

To account for noise when identifying clusters of homogenous patients, the authors applied the fuzzy *k*‐medoids algorithm to the obtained functional coefficients. By the B‐spline basis system, these coefficients allowed continuous smoothing functions to be found, synthesising the general trend of the observed data.^23^

#### 3.1.2 MASK‐air^®^

MASK, the Phase 3 ARIA (Allergic Rhinitis and its Impact on Asthma) initiative, is a Good Practice of DG Santé for digitally‐enabled, patient‐centred care.^65^, ^66^ It aims to improve the management of AR and asthma multimorbidity in a patient‐centred approach and to facilitate shared decision‐making.^67^ It includes (i) a freely available app (MASK‐air^®^, formerly the Allergy Diary, Android and iOS),^68^ operational in 28 countries and 20 languages, (ii) an interoperable electronic decision support system for the support of healthcare professionals in shared decision making,^69^ (iii) a web‐based interoperable questionnaire for physicians,^70^ (iv) the CARAT questionnaire for screening allergic diseases and assessing their control,^71^, ^72^ the European Quality of Life 5 Dimensions (EQ‐5D) and the Work Productivity and Activity questionnaire for asthma and (v) a sentinel network for air quality and pollen seasons.^73^ The TRL has been assessed for the MASK‐air^®^ app by MASK‐air^®^ members (TRL9).^74^

MASK‐air^®^ is CE1 registered and follows the GDPR.^60^, ^61^ It is in the process of being registered as a Medical Device Regulation Class 2. It operates under French jurisdiction. Geolocation is optional.

##### Published methodologic assessment of MASK‐air^®^

Following the COnsensus‐based Standards for the selection of health Measurement INstruments guidelines,^75^, ^76^, ^77^ internal consistency (Cronbach's α‐coefficient and test‐retest), reliability (intraclass correlation coefficients), sensitivity and acceptability of the MASK‐air^®^ visual analogue scales (VASs) were shown for global allergy symptoms, nose, eye and asthma.^32^ A subsequent study also concluded that VASs display high intra‐rater reliability, high test‐retest reliability, moderate/large responsiveness and moderate/high concurrent validity.^78^ The quality of MASK‐air^®^ data was checked^39^ by estimating the intra‐individual response variability index, a flexible way of detecting insufficient effort responding.^79^, ^80^ The independency of VAS questions (from each other) was confirmed using the Bland and Altman regression analysis.^29^, ^81^ Minimal Important Difference (MID) has been provided for most patient‐reported outcome measures (PROMs).

Most MASK‐air analyses were observational and non‐interventional. However, there was a clinical trial^82^ and a quasi‐experimental study.^83^

##### Acceptability of MASK‐air^®^ by patients

Many patients do not understand the needs and benefits of mHealth and may worry about data privacy. A minority may have difficulties in using a mobile phone. On the other hand, many patients over‐rely on internet‐based information and untested mHealth solutions. Two qualitative studies enabled a better understanding of the patients' needs and expectations,^74^ which permitted an according modification of the app. A study in Puglia (Italy) showed that older adults with a low level of education were able to use the MASK‐air^®^ app after a short training session.^84^

Overall, MASK‐air^®^ aims (i) to strengthen the EU digital single market,^85^ (ii) to develop the implementation of digitally‐enabled real‐life care pathways at the global level with the Global Alliance against Chronic Respiratory Diseases (GARD) and WHO^86^ and (iii) to develop a change management strategy in allergic and airway diseases.^87^

#### 3.1.3 Pollen App: Patient's hay fever diary

A model of individualised prediction of AR symptoms, named the Patient's Hay fever Diary (PHD), has been developed in Austria.^46^, ^47^, ^48^, ^54^, ^88^, ^89^, ^90^, ^91^, ^92^ There was a precise validation of aerobiologic data and of some clinical data in pollen allergic individuals. There were also numerous publications using the symptom data retrieved by this app. The Patient's Hay fever Diary was first available as a website in 2009 and was later included in Pollen App as well as in Husteblume, a spin‐off app. The system allows the documentation of allergy symptoms and the use of medication. It offers a simple comparison of personal symptoms with the regional pollen load for every user. Pollen App is available in eight countries/regions and six languages. The download and usage of this app are free of charge and there are no advertisements. Both systems adhere to the GDPR (Directive 95/46/EC) and collect only a minimum amount of personal data.^43^

Scientific studies using PHD/Pollen App symptom data first undergo a filtering process to assure the inclusion of exclusively qualitative data (e.g., for more seasons, with a certain number of entries per user) leading to robust results.^36^, ^37^, ^43^, ^81^, ^82^ The datasets have been analysed using not only statistical methods but also computational intelligence methods like Self Organising Maps.^36^, ^37^, ^43^, ^82^

Pollen App consists of three main parts: information, symptom documentation and medical assistance. Information is given concerning the pollen load including a personal allergy risk, the daily pollen load for various aeroallergens, forecast maps based on different models, as well as a dictionary with information on the most important allergenic plants. Symptom documentation is made in the pollen diary (Patient's Hay fever diary) and adapts the forecasts automatically if used (personal pollen information and allergy risk).^93^ Medical assistance concerns information on doctors in the vicinity, therapy recommendation situations for no‐, low‐, medium‐ and high‐risk burden as well as a symptom report (available since 2021) that can be shared with the patient's doctor.

Forecast data were part of the scientific research besides the exploitation of the symptom data. Nine freely available apps delivering pollen information and pollen forecasts had been tested with a focus on their prediction of the pollen load in the 2016 grass pollen season (Table 3).^48^ For six apps, the rates of correct pollen forecasts were around 50%, with Pollen App displaying the highest. Only two apps provided sufficiently accurate forecasts for the “readiness to flower” for grasses.

**TABLE 3:** Apps providing pollen forecasts assessed by Bastl et al.^48^ (information retrieved from their study)

App	Assessed city	Exact rates of correct pollen forecasts	Forecast on the readiness to flower
Pollen	Vienna	62.9	Yes
Biowetter	Vienna	31.8	No
Pollenwarner	Vienna	34.0	No
DWD	Berlin	41.1	No
Allergiehelfer	Berlin	48.6	No
Pollenflug	Berlin	50.7	No
Allergohelp	Berlin	45.2	No
Pollen news	Basel	42.4	Yes
Hayfever	London	35.7	No

A detailed description of the studies assessing PHD data is available in Section 4.1 of this review.

#### 3.1.4 Husteblume mobile phone health app

Husteblume is a mobile phone health app, developed in Germany, with the aim of facilitating the self‐management of pollen‐related AR. It includes providing information on the drugs most frequently used by patients with similar profiles. This app is a spin‐off of Pollen App. It used Patient's Hay fever Diary for symptom documentation and the forecasts of Pollen App. A study assessed the usability, changes in quality of life, health literacy and self‐efficacy for managing one's chronic disease. A total of 661 app users were included and 143 were evaluated after the pollen season.^55^ The patients using the app for a longer period perceived many subjective improvements, including better information about their allergy, improved quality‐of‐life and improved coping with their allergy.

#### 3.1.5 AirRater app

AirRater provides environmental data for patients with AR and allows them to record their symptoms and medication use. This app is available in English and is particularly tailored for Australia. A study assessed AirRater users by means of semi‐structured interviews, with most of them indicating that information provided by the app helped them to make decisions and implement behaviours to protect their health.^26^, ^52^

#### 3.1.6 AllerSearch

AllerSearch comprises a hay fever daily questionnaire, an assessment of the impact of rhinitis symptoms on work productivity and information on pollen levels. In addition, it is set to implement an artificial intelligence system to determine the degree of rhinitis severity based on photos from the eyes of the patients. A crowd‐sourced study using the smartphone app AllerSearch was carried out.^56^, ^57^ In 11,248 subjects (of whom 9041 had AR), demographic factors and symptoms associated with AR were assessed. In addition, using AllerSearch, clusters of patients with allergic rhinitis were obtained based on their presented symptoms.

### 3.2 Rhinosinusitis

There is only one mobile app within the defined criteria for CRS, namely mySinusitisCoach, an app for CRS patients. A study reported the cross‐sectional evaluation of the data of 626 users of this app.^58^, ^59^ Patient characteristics were analysed as well as the level of disease control based on the VAS global CRS symptom score and on adapted European Position Paper on Rhinosinusitis and Nasal Polyps (EPOS) criteria.^58^

## 4 ADDED VALUE OF mHEALTH APPS IN ALLERGY PHENOTYPING

Apps can be useful for generating hypotheses, which frequently need to be confirmed by more ‘classical’ clinical studies. An example of digital health in phenotype discovery was proposed by MASK‐air^®^.^40^, ^74^ Multimorbidity in allergic airway diseases was well known, but no data existed regarding the daily dynamics of symptoms. Using the MASK‐air^®^ app, eight hypothesis‐driven patterns were defined based on “Low” and “High” VAS levels. Days with rhinitis alone had the lowest VAS global allergy symptoms. A novel and previously unrecognised extreme pattern of uncontrolled multimorbidity was identified in 2.9% of the days: Rhinitis High ‐ Asthma High ‐ Conjunctivitis High. This hypothesis‐generating study was confirmed by classical epidemiologic studies,^94^, ^95^, ^96^ showing that it is important to consider ocular symptoms in severe asthma^95^ and that the severity of individual allergic diseases increases with the number of allergic morbidities.^97^ These findings were reinforced using computational analyses suggesting that there are common pathways in multimorbidity.^98^, ^99^ These were confirmed by a genomic approach in the MeDALL study (Mechanisms of the Development of Allergy, FP7)^100^ and showed a novel whole blood gene expression signature for asthma, dermatitis and rhinitis multimorbidity in children and adolescents.^101^

## 5 OPTIMISATION OF THE IDENTIFICATION OF THE POLLEN SEASON AND ROLE OF AIR POLLUTION

The definition of a pollen season determines the start and the end of the time period with a certain amount of pollen in the ambient air. Although common definitions should be used, different pollen season definitions were used for a long time, based on different terms and methods. Recently, suggested pollen season definitions for clinical trials were tested using apps and were applied for the first time to more aeroallergens.^88^, ^91^, ^92^ Clinical trials with pollen allergic patients need validated, high‐quality pollen data and forecasts to yield comparability and adhere to scientific standards.^102^

### 5.1 Pollen App (Patient's hay fever diary)

Using Pollen App, the representativeness of pollen concentrations was assessed for 20 pollen types in 2015–2016 in Vienna for rooftop and ground level. Comparisons were then performed with weather and symptom data.^90^ Computational intelligence methods were used to describe similarities and interdependencies, while the random forest algorithm was used to model symptom data. Most of the examined taxa showed similar patterns (e.g., *Betula*), while some showed differences at different heights (e.g., the Poaceae family). Some findings contradicted the literature and led to the posing of new hypotheses (e.g., concerning the abundance of Urticariaceae pollen in rooftop and ground levels). Temperature and humidity influenced daily pollen concentrations for most of the taxa. The rooftop trap was adequate when compared with the symptoms, justifying the recommendations concerning the location of a pollen trap and showing the importance of validations using symptom data.

The connection between pollen concentrations and crowd‐sourced symptom data provided new insights from daily and seasonal symptom load index data from 2013 to 2017 in Vienna.^47^ The Daily Symptom‐Load Index (SLI) and pollen concentration data were correlated. This study showed a linear relationship between SLI and pollen concentrations/seasonal pollen index daily but not on a seasonal basis. Cross‐reactivity to other pollen types, allergen content and air pollution could play a considerable role.

Quantification of the burden of pollen allergy was performed in Austria and Germany over 10 years using electronically‐generated symptom data from PHD.^54^ Four different symptom score calculation methods were applied to the datasets. This study did not detect significant differences between the various methods of symptom score calculation. Nasal symptoms determined about 40% of the scores.

Grass pollen‐triggered allergic symptoms vary within the season.^89^ Symptoms were studied in Vienna (Austria) during the 2014, 2015 and 2016 grass pollen seasons. They were compared with the grass pollen season defined either by grass pollen level data or phenology (grass species determination in the field). The symptom peak of most users was observed in the second section of the grass pollen season (70%), followed by the first section (20%) and the third section (10%). Differences between grass species were found.

### 5.2 Disentangling polysensitisation: The @IT.2020 study

An adequate definition of pollen seasons (e.g., regarding their beginning and end) is essential for optimal identification and management in AR patients.^103^, ^104^ A position paper by the European Academy of Allergy and Clinical Immunology (EAACI) proposed pollen season definitions for Northern and Central Europe. In the @IT.2020 multi‐centre study, pollen counts for many species were collected over 1 year (2018) in six Mediterranean cities (of four different countries) for seven pollen taxa (Poaceae, Oleaceae, Fagales, Cupressaceae, *Parietaria*, *Ambrosia* and *Artemisia*).^50^ The @IT.2020 study showed heterogeneous results between locations in terms of pollen species and duration of pollen seasons. A fragmentation of pollen seasons was found, with high pollen counts being separated by periods of low pollen counts.

In the Mediterranean area, patients with pollen‐induced AR are often polysensitised, rendering their assessment complex for aerobiologists and physicians. AllergyMonitor^®^ was used to improve the precision of diagnosing pollen allergy using daily symptom monitoring and graphical representations of airborne pollen data.^47^ Unfortunately, diagrams illustrating daily pollen concentrations from many sources in parallel make the interpretation of each of these curves difficult. This problem may be solved by using curves based on the cumulative transformation of pollen data using AllergyMonitor^®^.^105^

### 5.3 Impact of air pollution on rhinitis

Several studies have suggested an interaction between air pollution and pollen exposure, with an impact on allergy symptoms. However, large studies with real‐life data have not been available until recently.

In the POLLAR study,^41^, ^106^ associations between ozone and particulate matter with a diameter of <2.5 μm (PM_2.5_) and AR control assessed using MASK‐air^®^ were studied during grass and birch pollen seasons as well as outside the pollen season. Pollutant levels were assessed using the System for Integrated modeLing of Atmospheric coMposition (SILAM) database. Associations between ozone and uncontrolled rhinitis were found to be stronger during the grass pollen season than during the birch pollen season, possibly related to the relationship between ozone and higher temperatures.

Associations between six pollen types and respiratory symptoms were studied using the AirRater smartphone app in Tasmania (Australia) from 2015 to 2019. Associations between daily respiratory symptoms and pollen concentrations were first studied using Poisson regression models, with the case time‐series approach designed for app‐sourced data. Potentially non‐linear and lagged associations were examined with total pollen and six pollen taxa, with adjustments for seasonality and meteorology, and testing for interactions with particulate air pollution (PM_2.5_). Non‐linear associations were found between total pollen or individual pollen taxa and respiratory symptoms.

Using Pollen App, associations between symptoms, grass, birch or ragweed pollen levels, air quality and meteorological data (temperature, relative humidity) were studied for the metropolis of Vienna.^46^ Only ozone was significantly associated with symptom scores in birch, grass and ragweed pollen seasons. Further analyses in a model with meteorological data showed that the effect estimates of ozone were attenuated, but remained significant for the grass pollen season.

## 6 ADVANCES IN DIAGNOSIS

### 6.1 Aetiological diagnosis of seasonal allergic rhinitis

The analysis of the AR symptom severity scores during pollen exposure can be used to evaluate the clinical relevance of a patient's sensitisation to specific pollen. The comparison of symptom severity scores (Rhinitis Total Symptom Score, RTSS, in AllergyMonitor^®^) or of symptom scores (Pollen App) with pollen concentration data may guide the physician in the choice of the correct AIT composition.

### 6.2 Disease severity scores

#### 6.2.1 Comparison between symptom scores by AllergyMonitor^®^

Using AllergyMonitor^®^, 105 children with pollen allergy monitored their daily symptoms for 2 months during the grass pollen season. Six AR severity scores were compared with pollen counts at both population and individual levels^24^: (i) the RTSS, (ii) the Adjusted RTSS calculated based on the last observation, (iii) the Adjusted RTSS calculated based on the worst observation, (iv) the Rhino‐conjunctivitis Allergy‐Control‐SCORE, (v) the Average Combined Score and (vi) the average Adjusted Symptom Score.^24^ These disease severity scores tended to provide similar results at population level but often produced heterogeneous slopes in individual patients.

#### 6.2.2 Finding an optimal combined symptom‐medication score

There was an urgent need for a validated combined symptom‐medication score (CSMS) in AR, both for clinical practice and clinical trials. The CSMS needed to be developed against a gold‐standard tool/measurement that does not simply measure allergy symptoms or use of allergy medications and that assesses, if possible, a variable related to the economic impact of AR. Such tools include, among other endpoints, work productivity and quality‐of‐life for AR. Only MASK‐air^®^ and AllergyMonitor^®^ currently have these capabilities and can be used. However, the latter was missing EQ‐5D and Work Productivity and Activity Impairment (WPAI‐AS) and therefore MASK‐air^®^ was selected. Pollen App and PHD ask for quality of life, but data concerning this aspect have not yet been published.

The results showed that (i) A hypothesis‐driven score based on MASK‐air^®^ data was highly correlated with all instruments of quality‐of‐life and work tested, and had high concurrent validity and test‐retest reliability; (ii) Several data‐driven scores, in particular those based on cluster analyses, had a slightly higher level of correlation with identified endpoints; (iii) These results have been found to be highly reproducible across all tested regions (nine countries).^44^

### 6.3 Indication of allergen immunotherapy

The efficacy of AIT depends on the precise identification of the triggering allergen. However, diagnostics based on retrospective clinical history and sensitisation to whole extracts often lead to equivocal results. A study assessed a recently established algorithm for a clinical decision support system (@IT2020‐CDSS) for pollen rhinitis and its diagnostic steps (anamnesis, skin prick test or serum‐specific IgE, component‐resolved diagnosis and real‐time digital symptom recording by the AllergyMonitor^®^ eDiary) on doctors' AIT prescription decisions.^107^ After educational training on the @IT2020‐CDSS algorithm, 46 doctors (18 allergy specialists and 28 general practitioners) proposed a hypothetical AIT prescription for 10 clinical index cases. Decisions were recorded repeatedly, based on different steps of the algorithm. The combined use of the component‐resolved diagnosis and of the AllergyMonitor^®^ eDiary increased the hypothetical AIT prescriptions in both groups. Physicians considered the algorithm useful for the optimisation of classical diagnostic work‐up.

## 7 EVOLUTION IN MANAGEMENT

### 7.1 Medications

mHealth can be used to generate innovative insights into optimising treatment for the improvement of AR control. Two MASK‐air^®^ cross‐sectional real‐world observational studies were undertaken in 22 countries to complement a pilot study^36^ and provide novel information on medication use, disease control and work productivity in the everyday life of patients with AR.^38^, ^40^ The four most common intranasal medications containing intransal corticosteroid (INCS) (including INCS + intranasal antihistamine) and eight oral H_1_‐antihistamines (OAH) were studied. A total of 9122 users filled in 112,054 days of VASs up to 2017. The control of days with rhinitis using VAS was (i) similar for ‘no treatment’ and monotherapy with INCS and Azelastine‐Fluticasone intranasal formulation (best control), (ii) worse for monotherapy with OAH and (iii) the worst for multiple treatments (co‐medication). These observational studies using a very simple daily assessment tool (VAS) on a mobile phone answered questions previously thought infeasible.

### 7.2 Allergen immunotherapy

**FIGURE 1:**
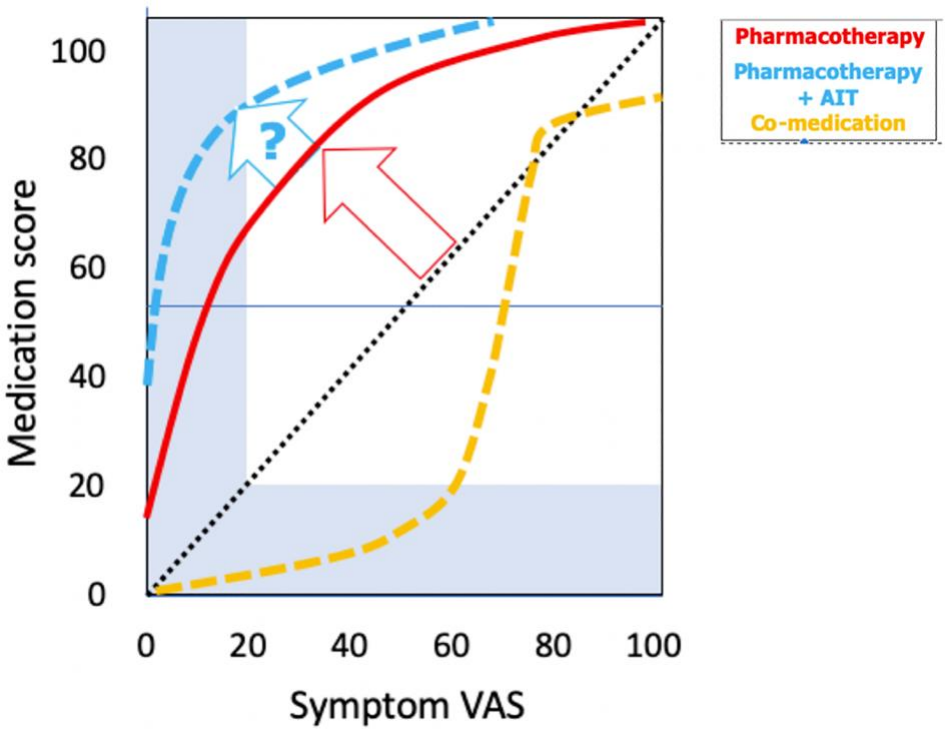
Impact of allergen immunotherapy (AIT) on real‐life. MASK data have shown that medications are more effective on days with low symptoms than on days with high symptoms (^____^). AIT improves symptoms for any level of medication (‐‐‐‐).

Real‐world data are available for AIT in the MASK‐air^®^ database.^108^ A proof‐of‐concept study has compared days of participants with AIT versus days of participants without AIT on VAS global allergy symptoms and VAS work. A total of 317,176 days were analysed, of which 11.4% involved AIT users. Lower median VAS global allergy symptoms and VAS work levels were observed for participants under AIT and were compared to the levels on days without treatment, with monotherapy or with polytherapy. This enabled us to better understand the role of AIT in real life (Figure 1). Nevertheless, further studies are required.

## 8 ADHERENCE TO TREATMENT IN ALLERGIC RHINITIS

### 8.1 Understanding adherence

mHealth may help to better understand adherence to treatment.^109^ Following a pilot study in less than 3000 AR patients,^36^ an observational cross‐sectional study was carried out on all MASK‐air^®^ users. The modified Medication Possession Ratio (MPR) and the Proportion of Days Covered (PDC) approach were used to assess secondary adherence.^110^ In total, 6949 users reported at least one VAS data recording, among whom 1195 were included in the analysis of adherence. Of the users, 11.3% were adherent to medication (MPR ≥70% and PDC ≤1.25), 4.2% were partly adherent (MPR ≥70% and PDC = 1.50) and 176 (14.6%) were switchers. On the other hand, 69.1% of the users were non‐adherent to medications (MPR ≤70%), indicating that adherence to AR treatment is low. This study proposed an approach for measuring retrospective adherence based on an app, representing a novel approach for analysing the behaviour of medication‐taking in a real‐world setting.^111^

### 8.2 Improving adherence

mHealth may improve adherence to treatment in chronic diseases. Children and adolescents (5–18 years) with moderate‐to‐severe seasonal AR to grass pollen, requiring a daily INCS administration, were recruited in April 2013.^22^ Participants were randomised to AllergyMonitor^®^ or to usual care (no diary) and followed up until 15 June 2013. Intra‐nasal mometasone use, expressed as both optimal adherence rate and average daily use, was higher in the AllergyMonitor^®^ group than in usual care. Disease knowledge improved among the patients using AllergyMonitor^®^ but not among the controls. However, no differences were observed at baseline and at follow‐up visits in the reported severity of disease, nasal flow and quality of life. This was due to an unexpected low temperature and pollen exposure during the observation period.

In another study on AllergyMonitor^®^ in 67 patients, the adherence to daily symptom monitoring remained high (>80%) throughout several weeks when prescribed and thoroughly explained by the treating doctor. Furthermore, app use was associated with improved adherence to symptomatic drugs and AIT.^21^

## 9 ASSESSMENT OF THE ECONOMIC BURDEN OF AR AND COST‐EFFECTIVENESS OF MANAGEMENT STRATEGIES

Allergic rhinitis is a burdensome condition, with an important impact on work^112^ and school productivity.^113^

MASK‐air^®^ can be used to quantify this impact, as it includes questions assessing the daily impact of AR symptoms on work productivity (VAS Work) and on school performance. In addition, the WPAI‐AS – which quantifies the impact of allergy on work and activities – can be answered optionally in MASK‐air^®^. MASK‐air^®^ enables the estimation not only of indirect costs resulting from loss of work productivity, but also of direct costs resulting from AR medication and AIT use. A monthly question asking the user whether he/she had an outpatient visit related to AR during the previous month could help to further improve the estimation of direct costs related to AR.

MASK‐air^®^ also includes EQ‐5D, whose scores can be converted into utilities (standardised measures of preferences that patients have for health status) for many of the countries where MASK‐air^®^ is available.^114^ Such a feature may be particularly useful for performing cost‐utility analyses, in which interventions are compared regarding their costs and also their effectiveness adjusted for patients' preferences.

## 10 NEXT‐GENERATION CARE PATHWAYS FOR THE DIGITAL TRANSFORMATION OF HEALTH CENTRED AROUND THE PATIENT

As an example of chronic disease care, MASK, in collaboration with professional and patient organisations in the field of allergy and airway diseases, proposes real‐life care pathways centred around the patient with AR and/or asthma multimorbidity.^115^ It uses mHealth to monitor environmental exposure^116^ and to sustain Planetary Health.^117^, ^118^, ^119^ Next‐generation guidelines have been proposed to assess the recommendations of Grading of Recommendations, Assessment, Development and Evaluation guidelines in AR and asthma using real‐world evidence and real‐world data obtained through mobile technology.^120^ Moreover, mHealth should be considered in the wider frame of Planetary Health.^117^

## 11 CONCLUSIONS

This review has provided several examples of how mHealth apps can play a key role in the scientific investigation and clinical assessment of AR and CRS. While mHealth apps may be a useful complementary tool in the diagnosis and management of patients with AR or CRS, some gaps still merit attention and should be the focus of future studies. Few apps included multimorbidity.

An important limitation of mHealth apps consists of the fact that only a minority of patients use them regularly. Identifying the patients who most probably use them more regularly would maximise their effectiveness. Such an identification could stem from a simple baseline questionnaire, whose development and validation should be the target of future studies.

On the other hand, connecting daily apps to medications (e.g., inhalers) or diagnostic tests (e.g., spirometry for asthma) may open the possibility of a more personalised monitoring of patients with AR or CRS. Furthermore, personalisation increases motivation to continuously use mHealth and eHealth apps and may improve adherence.

Other issues meriting discussion concern their certification, reimbursement, interoperability and quality control. Only a small fraction of available apps have published scientific results, with the content veracity of the remaining ones pending assessment.

Among the 1500 apps retrieved for AR and the several hundred retrieved for CRS, only a handful were selected for review, including three multilingual apps and two using a single language in AR. That is, while there are several apps claiming to be health‐related, only a few have been studied in a relevant manner, prompting the need for some quality control over health‐related apps. This may not only concern AR and CRS but also other chronic diseases.

Apps studied in a relevant manner were found to be of interest in the diagnosis, management and cost‐effectiveness of AR, as attested by the several examples presented in this review. In CRS, only one app has published results (and in only one single paper). There is an urgent need to validate other apps.

## AUTHOR CONTRIBUTIONS

Bernardo Sousa‐Pinto and Jean Bousquet wrote the paper. Rita Amaral, Aram Anto, Katharina Bastl, Uwe Berger, Markus Berger, Stephanie Dramburg, Oliver Pfaar, Ludger Klimek, Paolo M. Matricardi, Alessandro Travaglini and Salvatore Tripodi participated in the redaction of the paper for their own chapter and revised the paper. Marek Jutel, Wienczyslawa Czarlewski, Anna Bedbrook, Arunas Valiulis, Ioana Agache, Ignacio J. Ansotegui, Karl C. Bergmann, Sinthia Bosnic‐Anticevich, Fluvio Braido, Luisa Brussino, Victoria Cardona, Thomas Casale, G. Walter Canonica, Lorenzo Cecchi, Denis Charpin, Tomás Chivato, Derek K. Chu, Cemal Cingi, Elisio M. Costa, Alvaro A. Cruz, Philippe Devillier, Stephen R. Durham, Motohiro Ebisawa, Alessandro Fiocchi, Wytske J. Fokkens, Bilun Gemicioğlu, Maia Gotua, Maria‐Antonieta Guzmán, Tari Haahtela, Juan Carlos Ivancevich, Piotr Kuna, Igor Kaidashev, Musa Khaitov, Violeta Kvedariene, Désirée E. Larenas‐Linnemann, Brian Lipworth, Daniel Laune, Mario Morais‐Almeida, Joaquim Mullol, Robert Naclerio, Hugo Neffen, Kristoff Nekam, Marek Niedoszytko, Yoshitaka Okamoto, Nikolaos G. Papadopoulos, Hae‐Sim Park, Giovanni Passalacqua, Vincenzo Patella, Simone Pelosi, Nhân Pham‐Thi, Ted A. Popov, Frederico S. Regateiro, Sietze Reitsma, Monica Rodriguez, Nelson Rosario, Philip W. Rouadi, Boleslaw Samolinski, Ana Sá‐Sousa, Joaquin Sastre, Aziz Sheikh, Charlotte Suppli Ulrik, Luis Taborda‐Barata, Ana Todo‐Bom, Peter Valentin Tomazic, Sanna Toppila‐Salmi, Ioanna Tsiligianni, Erkka Valovirta, Maria Teresa Ventura, Antonio A. Valero, Rafael José Vieira, Dana Wallace, Susan Waserman, Sian Williams, Arzu Yorgancioglu, Luo Zhang, Mihaela Zidarn, Josep M. Antó, Torsten Zuberbier and João A. Fonseca are members of the MASK Think‐Tank, participated in the redaction of the paper and revised the paper.

## CONFLICTS OF INTEREST

Ignacio J. Ansotegui reports personal fees from Roxall, personal fees from UCB, personal fees from Faes Farma, personal fees from Sanofi, personal fees from Bial, personal fees from Abbott, personal fees from Bayer, personal fees from Organon, outside the submitted work. Sinthia Bosnic‐Anticevich reports grants from TEVA, personal fees from TEVA, personal fees from TEVA, personal fees from AstraZeneca, personal fees from AstraZeneca, personal fees from Boehringer Ingelheim, personal fees from Boehringer Ingelheim, personal fees from GSK, personal fees from Sanofi, personal fees from Mylan, outside the submitted work; Jean Bousquet reports personal fees from Chiesi, Cipla, Hikma, Menarini, Mundipharma, Mylan, Novartis, Purina, Sanofi‐Aventis, Takeda, Teva, Uriach, other from KYomed‐Innov, outside the submitted work. Victoria Cardona reports personal fees from ALK, personal fees from Allergopharma, personal fees from GSK, grants from Thermofisher, outside the submitted work. Lorenzo Cecchi reports personal fees from Thermofisher, personal fees from Sanofi, personal fees from Astra Zeneca, personal fees from Novartis, outside the submitted work. Alvaro A. Cruz reports personal fees from AstraZeneca, personal fees from Boehringer‐Ingelheim, personal fees from CHIESI, personal fees from GSK, personal fees from SANOFI, personal fees from Novartis, personal fees from EUROFARMA, personal fees from Abdi‐Ibrahim, outside the submitted work. Philippe Devillier reports personal fees and non‐financial support from ALK_Abello, personal fees and non‐financial support from Stallergenes Greer, personal fees and non‐financial support from Astra Zeneca, personal fees and non‐financial support from Chiesi, personal fees from GlaxoSmithKline, personal fees from Sanofi, personal fees and non‐financial support from Mylan/Meda Pharma, personal fees and non‐financial support from Boehringer Ingelheim, personal fees and non‐financial support from Novartis, personal fees and non‐financial support from Menarini, outside the submitted work. Stephen R. Durham reports personal fees from ALK, personal fees from Revelo, personal fees from Angany, personal fees from Stallergenes, personal fees from Abbott, outside the submitted work. Motohiro Ebisawa reports personal fees from Mylan, personal fees from ARS Pharmaceutical, personal fees from Novartis, outside the submitted work; João A. Fonseca reports he is a co‐founder of a company that develops mHealth technologies. Tari Haahtela reports other from GSK, Orion Pharma, and Sanofi, outside the submitted work. Juan Carlos Ivancevich reports personal fees from Laboratorios Casasco, personal fees from Faes Farma, personal fees from Abbott Ecuador, personal fees from Bago Bolivia, outside the submitted work. Joaquim Mullol reports personal fees from ALK‐Abello, personal fees from Allergopharma, personal fees from Stallergenes, personal fees from Anergis, personal fees from Allergy Therapeutics, personal fees from Leti, personal fees from HAL, during the conduct of the study; personal fees from GSK, personal fees from Novartis, personal fees from Teva, personal fees from Takeda, personal fees from Chiesi, outside the submitted work. Piotr Kuna reports personal fees from Adamed, personal fees from Berlin Chemie Menarini, personal fees from Boehringer Ingelheim, personal fees from AstraZeneca, personal fees from Glenmark, personal fees from Krka, personal fees from Novartis, personal fees from Polpharma, personal fees from GSK, personal fees from Sanofi, outside the submitted work. Violeta Kvedariene reports other from Norameda, other from BerlinCHemie Menarini, outside the submitted work. Désirée E. Larenas‐Linnemann reports personal fees from ALK, Allakos, Amstrong, Astrazeneca, Chiesi, DBV Technologies, Grunenthal, GSK, Mylan/Viatris, Menarini, MSD, Novartis, Pfizer, Sanofi, Siegfried, UCB, Alakos, Gossamer, Carnot, grants from Sanofi, Astrazeneca, Lilly, Pfizer, Novartis, Circassia, UCB, GSK, Purina institute., outside the submitted work. Brian Lipworth reports personal fees from Glenmark, grants and personal fees from Mylan, grants from Sanofi, from AstraZeneca, from null, from null, during the conduct of the study; and Son of BJL employee of AstraZeneca. Paolo Maria Matricardi reports personal fees from TPS Productions, outside the submitted work. Joaquim Mullol reports personal fees and other from SANOFI‐GENZYME & REGENERON, personal fees and other from NOVARTIS, personal fees and other from ALLAKOS, grants and personal fees from MYLAN Pharma, grants and personal fees from URIACH Group, personal fees from Mitsubishi‐Tanabe, personal fees from Menarini, personal fees from UCB, personal fees from AstraZeneca, personal fees from GSK, personal fees from MSD, outside the submitted work. Robert Naclerio reports other from Sanofi, Regeneron, Lyra, Celgene, GSK, and AstraZeneca., outside the submitted work; Dr. Naclerio reports other from Sanofi, Regeneron, Lyra, Celgene, GSK, and AstraZeneca., outside the submitted work. Yoshitaka Okamoto reports personal fees from Torii Co.,Ltd., personal fees from ALK Co., Ltd., personal fees from Kirin Pharmaceutical Company, personal fees from Tanabe‐Mitsubishi Company, outside the submitted work.Nikolaos G. Papadopoulos reports other from Gerolymatos Int., other from Capricare, other from Nutricia, from ALK, Asit Biotech, AstraZeneca, Biomay, Boehringer Ingelheim, GSK, HAL, Faes Farma, Medscape, Menarini, MSD, Mylan/Meda, Novartis, Nutricia, OM Pharma, Regeneron, Sanofi, Takeda, outside the submitted work. Simone Pelosi reports personal fees from TPS Production, outside the submitted work; In addition, Dr. Pelosi has a patent PCT/IT2018/000119 pending. Oliver Pfaar reports grants and personal fees from ALK‐Abelló, grants and personal fees from Allergopharma, grants and personal fees from Stallergenes Greer, grants and personal fees from HAL Allergy Holding B.V./HAL Allergie GmbH, grants and personal fees from Bencard Allergie GmbH/Allergy Therapeutics, grants and personal fees from Lofarma, grants from Biomay, grants from Circassia, grants and personal fees from ASIT Biotech Tools S.A., grants and personal fees from Laboratorios LETI/LETI Pharma, personal fees from MEDA; Pharma/MYLAN, grants and personal fees from Anergis S.A., personal fees from Mobile Chamber Experts (a GA2LEN Partner), personal fees from Indoor Biotechnologies, grants and personal fees from GlaxoSmithKline, personal fees from Astellas Pharma Global, personal fees from EUFOREA, personal fees from ROXALL Medizin, personal fees from Novartis, personal fees from Sanofi‐Aventis and Sanofi‐Genzyme, personal fees from Med Update Europe GmbH, personal fees from streamedup! GmbH, grants from Pohl‐Boskamp, grants from Inmunotek S.L., personal fees from John Wiley and Sons, AS, personal fees from Paul‐Martini‐Stiftung (PMS), personal fees from Regeneron Pharmaceuticals Inc., personal fees from RG Aerztefortbildung, personal fees from Institut für Disease Management, personal fees from Springer GmbH, grants and personal fees from AstraZeneca, personal fees from IQVIA Commercial, personal fees from Ingress Health, personal fees from Wort&Bild Verlag, personal fees from Verlag ME, outside the submitted work; and member of EAACI Excom, member of ext. board of directors DGAKI; coordinator, main‐ or co‐author of different position papers and guidelines in rhinology, allergology and allergen‐immunotherapy. Sanna Toppila‐Salmi reports grants from GSK, personal fees from AstraZeneca, personal fees from ALK Abelló, personal fees from ERT, personal fees from GSK, personal fees from Novartis, personal fees from Sanofi Pharma, personal fees from Roche, outside the submitted work. Boleslaw Samolinski reports personal fees from Polpharma, personal fees from Viatris, grants and personal fees from AstraZeneca, personal fees from TEVA, personal fees from patient ombudsman, personal fees from Polish Allergology Society, grants from GSK, outside the submitted work; Joaquin Sastre reports grants and personal fees from SANOFI, personal fees from GSK, personal fees from NOVARTIS, personal fees from ASTRA ZENECA, personal fees from MUNDIPHARMA, personal fees from FAES FARMA, outside the submitted work. Ana Todo‐Bom reports personal fees from Astrazeneca, personal fees from GSK, personal fees from Novartis, personal fees from Bial, grants and personal fees from Leti, personal fees from Mylan, personal fees from AbbVie, grants and personal fees from Sanofi, outside the submitted work. Salvatore Tripodi reports personal fees from TPS Production, outside the submitted work; In addition, Dr. Tripodi has a patent PCT/IT2018/000119 pending. Ioanna Tsiligianni reports grants from Grants from Boehrniger Ingelheim, Glaxo Smithkline, Astra Zeneca and personal fees for advisory boards or speaker bureau from Astra Zeneca, Chiesi, Novartis, outside the submitted work. Charlotte Suppli Ulrik reports grants and personal fees from AZ, personal fees from GSK, grants and personal fees from BI, grants and personal fees from Sanofi, personal fees from Orion Pharma, personal fees from Pfizer, personal fees from TEVA, grants and personal fees from Novartis, outside the submitted work; Torsten Zuberbier reports personal fees from AstraZeneca, personal fees from AbbVie, personal fees from ALK, personal fees from Almirall, personal fees from Astellas, personal fees from Bayer Health Care, personal fees from Bencard, personal fees from Berlin Chemie, personal fees from FAES, personal fees from HAL, personal fees from Leti, personal fees from Meda, personal fees from Menarini, personal fees from Merck, personal fees from MSD, grants and personal fees from Novartis, personal fees from Pfizer, personal fees from Sanofi, personal fees from Stallergenes, personal fees from Takeda, personal fees from Teva, personal fees from UCB, grants from Henkel, personal fees from Kryolan, personal fees from L'Oréal, outside the submitted work; and Organisational affiliations; Commitee member: WHO‐Initiative ‘Allergic Rhinitis and Its Impact on Asthma’ (ARIA); Member of the Board: German Society for Allergy and Clinical Immunology (DGAKI); Board Chairman: European Centre for Allergy Research Foundation (ECARF); President: Global Allergy and Asthma European Network (GA2LEN); Member: Committee on Allergy Diagnosis and Molecular Allergology, World Allergy Organization (WAO).

## Data Availability

Data sharing is not applicable to this article as no new data were created or analysed in this study.
